# Effect of a specially formulated diet on progression of heart enlargement in dogs with subclinical degenerative mitral valve disease

**DOI:** 10.1111/jvim.16796

**Published:** 2023-07-01

**Authors:** Mark A. Oyama, Brian A. Scansen, Adrian Boswood, Guilherme Goldfeder, Steven Rosenthal, Richard Cober, Kendra LaFauci, Ryan C. Friese, Márcia Gomes, Yu‐Mei Ruby Chang, Qinghong Li

**Affiliations:** ^1^ School of Veterinary Medicine University of Pennsylvania Philadelphia Pennsylvania USA; ^2^ College of Veterinary Medicine & Biomedical Sciences Colorado State University Fort Collins Colorado USA; ^3^ Royal Veterinary College North Mymms UK; ^4^ School of Veterinary Medicine University of São Paulo São Paulo Brazil; ^5^ CVCA‐Columbia Columbia Maryland USA; ^6^ CVCA‐Annapolis Annapolis Maryland USA; ^7^ Petcardia Castle Rock Colorado USA; ^8^ College of Veterinary Medicine University of Illinois Urbana‐Champaign Illinois USA; ^9^ Nestlé Purina Research St. Louis Missouri USA

**Keywords:** clinical trial, echocardiography, fatty acids, metabolomics, myxomatous mitral valve disease

## Abstract

**Background:**

Previous studies in dogs with degenerative mitral valve disease (DMVD) have identified altered myocardial energy metabolism and oxidation, which might contribute to cardiac hypertrophy. Diets rich in medium chain fatty acids and antioxidants are a potential means of treatment. A previous clinical study found significantly smaller left atrial diameter (LAD) and left atrium‐to‐aorta diameter ratio (LA : Ao) in dogs with subclinical DMVD fed a specially formulated diet vs control diet for 6 months.

**Hypothesis/Objectives:**

A specially formulated diet will slow or arrest left heart enlargement in dogs with subclinical DMVD over 365 days.

**Animals:**

One hundred twenty‐seven dogs with unmedicated subclinical DMVD; 101 dogs in the per protocol cohort.

**Methods:**

Randomized double‐blinded controlled multicenter clinical trial.

**Results:**

The study's primary composite outcome measure was the sum of percentage change in LAD and left ventricular internal dimension at end‐diastole (LVIDd) at day 365. In the per protocol cohort, the outcome measure increased by 8.0% (95% confidence interval [CI], 2.9%‐13.1%) in dogs receiving the test diet vs 8.8% (95% CI, 5.1%‐12.5%) in dogs receiving control diet (*P* = .79). Neither component of the primary outcome measure was significantly different between groups (LAD, *P* = .65; LVIDd, *P* = .92). No difference was found in mitral valve E wave velocity (*P* = .36) or the proportion of dogs withdrawn from the study because of worsening DMVD and heart enlargement (*P* = .41).

**Conclusions and Clinical Importance:**

Feeding a specially formulated diet for 365 days was not associated with a significantly different rate of change of left heart size in dogs with subclinical DMVD as compared to control.

**Abbreviations**
2D: 2‐dimensional
ALT: alanine transaminase
AoD: aortic root diameter
CHF: congestive heart failure
CI: confidence interval
CKCS: Cavalier King Charles Spaniel
DICOM: Digital Imaging and Communications in Medicine
DMVD: degenerative mitral valve disease
E : A: E wave to A wave velocity ratio
ECL: echocardiographic core laboratory
Emax: maximum E wave velocity
FAs: fatty acids
FS: fractional shortening
IQR: interquartile range
IVSd: interventricular septum wall thickness at end‐diastole
LA : Ao: left atrial to aortic root diameter ratio
LAD: left atrial diameter
LOCF: last observation carried forward
LVIDd: left ventricular internal diameter at end‐diastole
LVIDs: left ventricular internal diameter at end‐systole
LVPWd: left ventricular posterior wall thickness at end‐diastole
MCT: medium chain triglycerides

## 1 INTRODUCTION

Degenerative mitral valve disease (DMVD) can result in progressive left heart enlargement as the severity of mitral regurgitation increases. Common measures of DMVD severity include the echocardiographic left atrial diameter (LAD), left atrium‐to‐aorta diameter ratio (LA : Ao), and the diameter of the left ventricle at end‐diastole (LVDd).^1^ The heart is a metabolically active organ and requires a constant supply of high energy phosphates. Long chain fatty acids (FAs) and glucose are the 2 most common myocardial energy substrates and undergo β‐oxidation or glycolysis, respectively.^2^ Limitations associated with β‐oxidation of long chain FAs include reliance on a carnitine‐mediated mitochondrial transport system and production of reactive oxygen species byproducts.^3^ Ischemia, uncoupling of glucose uptake and oxidation, insulin resistance, and high angiotensin II concentrations impair glycolysis and can lead to an energy starved state.^4^ Diet is a potential means of altering myocardial metabolism by selective avoidance or enrichment of certain ingredients. For instance, foods such as coconut and palm oil are rich in medium chain triglycerides (MCT), which are precursors of medium chain FAs. Medium chain FAs do not depend on mitochondrial carnitine transport and are easily oxidized as compared to long chain FAs.^5^ Foods rich in antioxidants, such as vitamin E or taurine, or rich in long chain polyunsaturated FA, such as omega‐3 FAs, similarly offer potential benefit. A previous controlled study^6^ reported the effect of a custom formulated diet, enriched with antioxidants, MCT, and other metabolic precursors, on echocardiographic left atrial size in 19 dogs with subclinical DMVD. Dogs receiving the test diet experienced significant 3% and 4% decreases in LAD and LA : Ao, respectively, compared to increases of 7% and 11% in dogs receiving control diet. This study was performed in a sample of primarily adult Beagles housed in a research environment for 6 months. We sought to determine the effect of a similarly formulated diet in a larger cohort of client‐owned dogs over a 12‐month feeding period. The hypothesis was that the test diet would slow or prevent echocardiographic left heart enlargement in dogs with mild subclinical DMVD as compared to a control diet over a 12‐month feeding period.

## 2 MATERIALS AND METHODS

The study was a multicenter prospective randomized double blinded clinical trial. The study was approved by the appropriate Institutional Animal Care and Use Committees, and informed owner consent was obtained. Inclusion criteria were as follows: ≥6 years of age, left‐sided systolic murmur grade ≥2, systolic blood pressure <180 mm Hg, color flow Doppler echocardiographic evidence of mitral regurgitation, and normalized M‐mode internal LV diastolic dimension (LVDdN) <1.7, and 2‐dimensional (2D) LA : Ao <1.6 as measured from the right short axis parasternal views. Exclusion criteria were as follows: receipt of cardiac medications or prohibited dietary supplements within the preceding 14 days, receipt of prescription diet for a preexisting condition, history of previous or current congestive heart failure (CHF), gastrointestinal signs, clinically relevant noncardiac disease or cardiac disease other than DMVD, or pulmonary hypertension defined as Doppler tricuspid regurgitation velocity ≥4.0 m/s or clinically relevant septal flattening or right ventricular concentric hypertrophy.

At time of screening, echocardiography, physical examination, indirect blood pressure measurement, CBC and serum biochemistry were performed. After screening, eligible dogs underwent a 2‐week diet run‐in period to test the acceptability of both study diets. Dogs were transitioned onto each of the diets, which each were fed for 5 days. The owner scored the palatability and tolerance of each diet using a prespecified scale (see Supplemental Information). The dog was required to eat both diets with the same enthusiasm, completeness, and overall acceptance without gastrointestinal signs (i.e., scores of 1) to successfully complete the run‐in and be randomized. Randomization was performed at the center level by breed (Cavalier King Charles Spaniel [CKCS] vs. non‐CKCS) and diet in blocks of 4. The study's test article was a specially formulated dry kibble diet enriched in MCT and antioxidants. The diets were manufactured at 3 separate plants, which supplied diet to the North American sites, Brazil, and the United Kingdom, respectively. The study diets were identical to each other in appearance and packaging and were labeled with 1 of 4 letters (A‐D) to help maintain double blinding throughout the study. The amount of diet fed was calculated based on the dog's ideal body weight and maintenance energy requirement. Owners were instructed that the study diet should comprise at least 90% of their dog's diet with the remaining 10% comprised of treats and other foods as long as prohibited dietary supplements were not fed. For reasons of other illness, such as dietary indiscretion or transient diarrhea or decreased appetite, study diet holidays of up to 4 consecutive days were permitted as long as the days off the study diet amounted to ≤16 days in total over the duration of the study. Recheck physical examination, echocardiography, serum electrolyte concentrations, renal function tests, and blood pressure were performed 180 and 365 days after randomization. Between these visits, recheck physical examination was performed 90 and 270 days after randomization. At each visit, the quantity of study diet being fed was reevaluated based on body condition, body weight, and discussion with the owner. Study personnel contacted owners by telephone or email every 45 days to ensure compliance with the diet feeding. Adverse events, whether or not thought to be related to the diet, were recorded and classified according to severity and their potential relationship to the diet.

**FIGURE 1:**
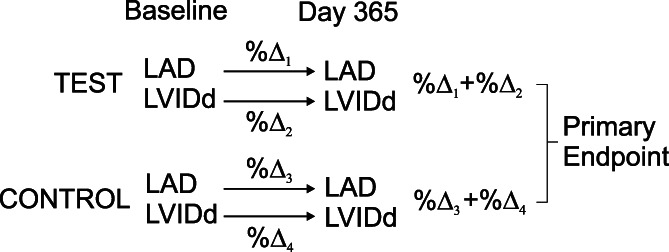
Construction of the composite primary outcome variable. Dogs were evaluated at baseline and day 365. The sum of the percentage change in the absolute values of left atrial diameter (LAD) and left ventricular internal diameter at end‐diastole (LVIDd) between the test and control cohorts was compared. Given the relatively slow and variable progression of degenerative mitral valve disease (DMVD) as well as the variability of echocardiographic measurements in dogs with subclinical DMVD, a composite endpoint was used to help maximize sensitivity to changes in left heart size.

The study's primary outcome measure was the difference in the sum of the absolute percentage change of LAD and LVDd from day 0 to day 365 between groups in the per protocol cohort (Figure 1). The outcome measure was independent of the dog's body weight or change in weight during the study. Sample size was calculated a priori based on a power of 80%, alpha of 0.05, and a hypothesized clinically relevant difference of ≥8% (SD, 14%) in progression of left heart size between groups (eg, control 8% increase vs. test diet 0% increase). A total of 98 dogs (49 in each group) were needed in the per protocol sample. To this total, 14 dogs were added to account for loss of power in the event the rate of disease progression was not normally distributed. An additional 10 dogs were added to account for withdrawals for a total recruitment target of 122 dogs.

The interobserver and intraobserver variability associated with echocardiography^7^, ^8^ tends to decrease study power. The study methods specifically included 3 design characteristics to increase study sensitivity and power. First, at each study timepoint, 2D, M‐mode, and color flow echocardiography exclusively was performed by a single board‐certified cardiologist (i.e., the site's primary investigator). Second, the study outcome measure used the sum of the LAD and LVDd as a composite measure of global left heart enlargement rather than either measure individually. Third, the primary outcome measure was determined by an echocardiography core laboratory (ECL) with 1 prospectively assigned investigator (BAS). The ECL prospectively established standard processes for image acquisition, transfer, and analysis that each site and investigator followed (see Supplemental Information). Briefly, LVDd and the LAD and aortic diameter (AoD) were measured from the right parasternal short axis M‐mode and 2D views, respectively. The LVDd was measured from the inner edge of the interventricular septum to the inner edge of the ventricular posterior wall. The AoD was measured from the inner edge of the midpoint of the right aortic sinus along the commissure of the left and nonadjacent sinuses to the point where the sinuses merged with the adjacent aortic wall. The LAD was measured beginning at the same point that the AoD line ended to the inner edge of the left atrial wall. Images and cine loops obtained by the site investigators were sent in Digital Imaging and Communications in Medicine (DICOM) format to the ECL for primary outcome measure analysis. The ECL blindly analyzed images using a vendor‐neutral image server and analysis package (TOMTEC‐ARENA version 2.41; TOMTEC Imaging Systems GmbH; Unterschleissheim, Germany). Measurements were performed at the end of the study to avoid temporal bias or drift. Measurements by the ECL were not used to make clinical decisions in real‐time. For this purpose, the site investigator used measurements performed on‐site to assess eligibility and inform clinical decision‐making during the study. In this manner, echocardiographic images were measured by 2 different individuals, the first being the study site investigator and the second being the ECL.

Dogs were withdrawn from the study for protocol violations, intolerance or refusal to consume the study diet, or need for prohibited medications or diets. Dogs withdrawn from the study were excluded from the per protocol sample during analysis. Secondary outcome measures were as follows: proportion of dogs withdrawn at day 180 so as to begin PO pimobendan administration because of the finding of LVDdN and LA : Ao ≥1.7 and ≥1.6, respectively, change in mitral E wave maximum velocity (Emax), and mitral E to A wave velocity ratio (E : A).

Statistical analysis was performed by a blinded independent statistician (YRC) who was not involved in hypothesis generation or clinical procedures. Data were assessed for normality using histograms and the Shapiro‐Wilk test. Between group comparisons were performed using unpaired 2‐tailed *t*‐tests, Wilcoxon rank sum tests, and Chi‐squared tests as appropriate. Linear mixed modeling was used to determine the potential effect of age and breed (i.e., CKCS vs non‐CKCS) on the primary outcome measure using center as a random effect. Stratified analysis was performed to evaluate the impact of geographic site location (i.e., North America and South America) on the effect of diet on the primary outcome measure. Box and whisker plots were created with the box encompassing the 25th to 75th percentile values, the line indicating the median value, and whiskers extending to the 5th and 95th percentile values. We tested the sensitivity of the primary outcome in 2 ways. First, we used the last observation carried forward (LOCF) from day 180 to day 365 in dogs that were withdrawn at day 180 as opposed to excluding them from the outcome analysis. Second, we examined the outcome using echocardiographic measurements performed on‐site by the investigators in place of the ECL measurements. Significance was defined as *P* < .05.

## 3 RESULTS

### 3.1 Study sample

**FIGURE 2:**
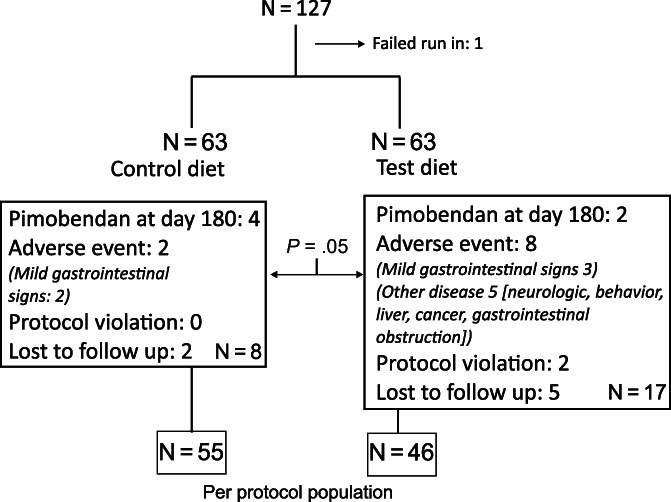
Flowchart of the study indicating the number of dogs randomized to each of the 2 study groups, number of dogs withdrawing from the study for various reasons, and composition of the per protocol sample. The per protocol sample included 55 dogs in the control group and 46 dogs in the test group for a total of 101 dogs.

One hundred twenty‐seven dogs were recruited from 8 sites between January 2018 and October 2020. Originally, 4 study sites (University of Pennsylvania, Royal Veterinary College, Colorado State University, and University of São Paulo) were involved. In 2019, the study was expanded to include the 4 additional sites (Petcardia, University of Illinois, CVCA‐Columbia, CVCA‐Annapolis) in order to accelerate the enrollment rate. The study flow diagram is shown in Figure 2. One dog did not successfully complete the run‐in period and 126 dogs were randomized to receive test diet (n = 63) or control diet (n = 63). A significantly higher number of dogs in the test diet cohort withdrew from the study as compared to the control diet cohort (17 vs 8; *P* = .05). This result primarily was associated with development of other systemic diseases or conditions believed to be unrelated to the diet. The per protocol cohort consisted of 101 dogs, including 46 dogs that received the test diet and 55 dogs that received the control diet (Table 1). Dogs typically were older male dogs of small to medium body size. The most common breed was the CKCS (29/101, [29%]) followed by mixed breed (12/101, [12%]), and Chihuahuas (10/101, [10%]). The demographics, blood pressure, and echocardiographic measurements (Table 1) as well as the hematology and serum biochemistry results (Supplemental Table S1) were similar between study groups at baseline.

**TABLE 1:** Demographic and physical and echocardiographic findings of the per protocol population as measured by the ECL.

	Control	Test	*P*
N	55	46	
Age (y)	10.5 (8.6‐11.1)	9.9 (8.1‐11)	.1
Sex (male/female)	35/20	30/16	.87
Body weight (kg)	8.2 (7.0‐9.7)	7.9 (5.8‐10.6)	.8
Breed (CKCS/non‐CKCS)	17/38	12/24	.66
Murmur grade (2/3/4/5/6)	29/18/6/1/1	23/16/6/1	.82
Systolic blood pressure (mm Hg)	143 (17)	144 (17)	.97
Normalized values^a^			
AoD	0.71 (0.66‐0.76)	0.72 (0.68‐0.76)	.51
LAD	1.00 (0.13)	1.00 (0.09)	.83
LVIDd	1.50 (0.16)	1.47 (0.12)	.38
LVIDs	0.84 (0.14)	0.84 (0.12)	.96
IVSd	0.45 (0.42‐0.48)	0.45 (0.41‐0.51)	.96
LVPWd	0.45 (0.05)	0.45 (0.06)	.77
Absolute values			
LAD (cm)	2.06 (0.33)	2.02 (0.29)	.51
AoD (cm)	1.46 (0.20)	1.46 (0.20)	.87
LVIDd (cm)	2.77 (0.37)	2.71 (0.37)	.42
LVIDs (cm)	1.63 (0.33)	1.62 (0.33)	.94
LA : Ao	1.42 (0.14)	1.39 (0.14)	.3
FS (%)	40.0 (36.1‐43.7)	40.0 (35.2‐44.1)	.64
Emax (m/s)	0.67 (0.57‐0.73)	.67 (0.59‐0.79)	.44
E : A	0.98 (0.83‐1.21)	1.1 (0.83‐1.23)	.32

*Note*: Data are displayed as mean (SD), median (IQR), or count.

Abbreviations: AoD, aortic root diameter; CKCS, Cavalier King Charles Spaniel; E : A, E wave to A wave velocity ratio; ECL, echocardiographic core laboratory; Emax, maximum E wave velocity; FS, fractional shortening; IQR, interquartile range; IVSd, interventricular septum wall thickness at end‐diastole; LA : Ao, left atrial to aortic root diameter ratio; LAD, left atrial diameter; LVIDd, left ventricular internal diameter at end‐diastole; LVIDs, left ventricular internal diameter at end‐systole; LVPWd, left ventricular posterior wall thickness at end‐diastole.

^a^ Normalized values derived from Cornell, Kittleson, Della Torre, et al. Allometric scaling of M‐mode cardiac measurements in normal adult dogs. *J Vet Intern Med*. 2004;18:311‐321.

### 3.2 Primary outcome measure

**FIGURE 3:**
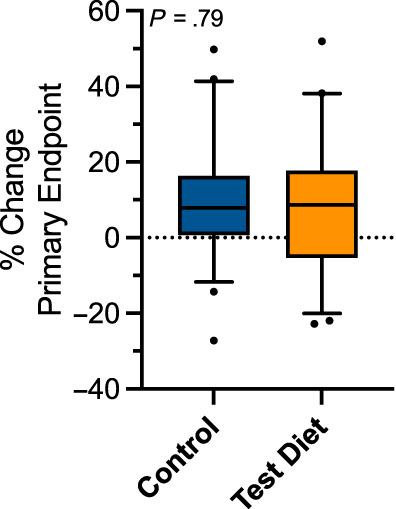
Percentage change in the primary outcome measure (ie, sum of the percentage change in the absolute values of left atrial diameter and left ventricular internal diameter at end‐diastole) at day 365 in the per protocol sample. The associated *P* value was .79.

**FIGURE 4:**
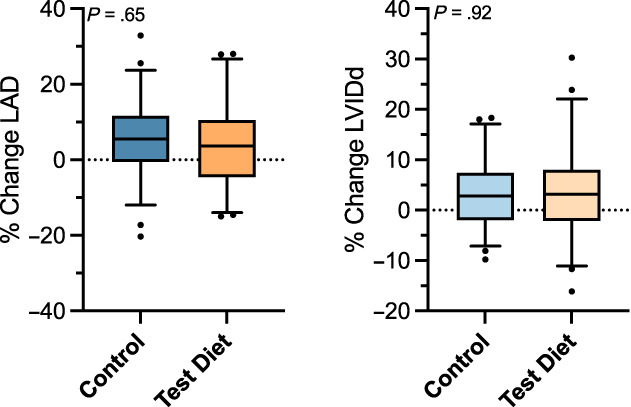
Percentage change of each component of the primary outcome measure at day 365 in the per protocol sample: (A) percentage change in left atrial diameter (LAD) with an associated *P* value of .65; (B) percentage change in left ventricular internal diameter at end‐diastole (LVIDd) with an associated *P* value of .92.

At day 365, dogs receiving the test diet experienced an average increase in the primary outcome measure of 8.0% (95% CI, 2.9%‐13.1%) as compared to 8.8% (95% CI, 5.1%‐12.5%) in dogs receiving the control diet (Figure 3). These differences were not significant between groups (*P* = .79). The rate at which progression of heart enlargement developed was normally distributed (Shapiro‐Wilk *P* = .31). Neither of the individual components of the primary outcome measure significantly differed between groups. Dogs receiving the test diet experienced a 4.4% (95% CI, 1.1%‐7.8%) increase in LAD as compared to 5.4% (95% CI, 2.7%‐8.1%) in dogs receiving the control diet (*P* = .65; Figure 4A). Dogs receiving the test diet experienced a 3.6% (95% CI, 0.84%‐6.3%) increase in LVDd as compared to 3.4% (95% CI, 1.6%‐5.3%) in dogs receiving the control diet (*P* = .92; Figure 4B). No significant effect of age (*P* = .21) or breed (*P* = .1) was found on the primary outcome measure. Results specific to sites in North America (*P* = .55) or South America (*P* = .13) were not significant. Analyses of the primary endpoint using LOCF data from day 180 (P = .6) as well as use of the site investigator's echocardiographic measurements in place of the ECL measurements (*P* = .91) were not significant.

### 3.3 Secondary outcome measures

The change in the primary outcome measure between 0 and 6 months was not significantly different between groups (test diet, 2.1%; 95% CI, −1.3% to 5.4% vs control, 4.5%; 95% CI, 0.89%‐8.1%; *P* = .33) nor was the change between 6 and 12 months (test diet, 5.3%; 95% CI, 1.6%‐9.0% vs control, 5.1%; 95% CI, 1.6%‐8.7%; *P* = .94). The change in Emax between baseline and day 365 was not significantly different between groups (test diet, 0.63%; interquartile range [IQR], −9.3% to 15.6% vs control, 5.7%; IQR, −5.6% to 12.9%; *P* = .36) nor was the change in mitral valve E : A (test diet, −6.2%; IQR, −12.3 to 10.3 vs control, 2.4%; IQR, −12.8% to 11.5%; *P* = .47). The proportion of dogs withdrawn at day 180 to start PO pimobendan was not significantly different between groups (test diet, 4/63 [6.4%] vs control, 2/63 [3.2%]; *P* = .41). Systolic blood pressure at day 365 as well as the difference in blood pressure at day 365 compared to baseline were not different between groups (day 365: control, 150 mm Hg [n = 54]; IQR, 130‐160 vs test diet, 144 mm Hg [n = 44]; IQR, 120‐150; *P* = .66; difference day 365 vs day 0: control, 1 mm Hg [n = 54; SD, 25] vs test diet, 2 mm Hg [n = 44; SD, 19]; *P* = .46). Significant but small and clinically irrelevant increases in alanine transaminase (ALT) activity and decreases in hematocrit as well as serum creatinine and potassium concentrations were observed in dogs receiving test diet vs control diet (see Supplemental Information Tables S1 and S2).

## 4 DISCUSSION

Our main finding was that feeding of a specially formulated diet for 365 days was not associated with prevention or slowing of rate of echocardiographic left heart enlargement in dogs with mild subclinical DMVD. Design of our study predated quantitative criteria distinguishing dogs with subclinical DMVD. By coincidence, our study and subsequent consensus guidelines^1^ chose to use previously reported LA and LV dimensions^9^ as the basis for study inclusion and definition of DMVD Stage B1, respectively. In light of the guidelines^1^ currently in use at the time of writing, our study population involved dogs with Stage B1 DMVD. Our findings are in contrast to those of a previous study^6^ in 19 dogs also with subclinical DMVD wherein dogs in the test diet group (n = 9) experienced a decrease in LA : Ao after 3 and 6 months and in LAD after 6 months as compared to the control group (n = 10). This particular study specifically motivated our larger study and the reasons for the discrepant results are not clear. The 2 studies are similar in several aspects, including the diet, subclinical stage of DMVD, and use of echocardiographic outcome measures, whereas the studies differ in other potentially important ways including breed, duration, size of study, and certain features of the study design. The studies were similar in their use of small to medium‐sized dogs with mild subclinical DMVD and little to no LA or LV enlargement. In our study, the mean baseline LA : Ao was slightly higher than in the previous study, which could be the result of small differences in study samples or measurement techniques. In our study, we included the cranial wall of the LA in the LAD measurement and measured the AoD along the zone of apposition between the left and noncoronary cusps of the aortic valve whereas the previous study measured the LAD from inner edge to inner edge and measured the AoD along the zone of apposition between the right and noncoronary aortic valve cusps. Both studies used paired observations to calculate percentage change between 2 study time points, and it is unlikely that the difference in measurement methods affected results.

The 2 studies also differed with regard to breed distribution, duration of follow‐up, and environment. Dogs in the previous study were housed at a single nutritional research center where breed, diet, living conditions, and other environmental characteristics were relatively controlled. The previous study involved 17 Beagles and 2 Miniature Schnauzers whereas our study involved only 1 Beagle, no Miniature Schnauzers, and a wide variety of other small and medium purebred and mixed breed dogs. Previous studies have reported differences between CKCS vs non‐CKCS breeds with respect to reference ranges of radiographic heart size, rate of DMVD progression, survival after heart failure, and other clinical outcome measures. In our study, we ensured an equal distribution of CKCS between the 2 study cohorts by including breed in the randomization scheme, and no significant differences in outcome measures were observed between CKCS and non‐CKCS breeds.

In previous studies,^6^, ^10^ a diet similar to our test diet was associated with significantly increased circulating MCT, omega‐6‐to‐omega‐3 FA ratio, and nitric oxide and carnitine biosynthesis precursors as compared to control diet. Our study protocol did not include metabolomic testing, which might have provided additional insights. In the previous studies, feeding of the dogs was strictly controlled. In our cohort of client‐owned dogs, we recognized the impossibility of excluding any and all foods other than the study diet for 365 days, and owners were allowed to provide small amounts of food other than the study diet and to take diet holidays. Although foods or supplements with potential to interfere with the study (e.g., fish oil, antioxidants, cardiac glycosides, other prescription diets) were strictly prohibited, we cannot rule out an effect of other foods.

The previous study^6^ lasted for 6 months with changes in LA : Ao seen as soon as 3 months and a significant interaction between diet and time points for both LA : Ao and LAD, but not for LVIDd, was observed. In our study, rates of LAD and LVIDd changes were the same between groups at both time points. Both studies recruited dogs with normal LAD and LVIDd chamber dimensions, implying the absence of clinically relevant volume retention and eccentric hypertrophy at baseline. In both studies, the control group experienced an increase in LAD and LA : Ao over baseline. In our study, LAD and LVIDd both increased to a similar extent in the test group as the control group. In the previous study, the test diet group experienced not just slowing or prevention of enlargement but a decrease in LAD compared to baseline, which is unusual given the stage of disease at baseline. Based on the results of our study, the findings of the previous study could have been a result of the relatively small study cohort.^11^

The many different metabolic processes within the heart are multifaceted and highly interwoven. Our study hypothesis partly was based on blood‐based metabolomic findings from previous studies, which included client‐owned study samples similar to ours.^12^, ^13^ Untargeted metabolomic data helps generate testable hypotheses, but the complexity of the metabolome might be such that simple interventions such as dietary long chain omega‐3 FAs or MCTs fail to account for important parts of the pathways that are yet unknown. The timing of metabolic changes and interventions also might play an important role. Previous studies^12^, ^14^ indicate that metabolic derangements in FA pathways worsen with increasing disease severity, and interventions might have importance in later stages. Thus, despite our results, study of the metabolome and diet interventions are potentially valuable. Dietary modification is relatively simple, inexpensive, and more widely available than conventional pharmaceutical approaches, which raises the potential value of this strategy.

The negative findings of our study prompt questions about study power. More dogs were withdrawn from the study than originally anticipated. The loss was mitigated by the fact that the rate of left heart enlargement was normally distributed. Our per protocol sample of 101 dogs exceeded the prespecified target of 98 dogs assuming these conditions. A significantly higher number of dogs in the test group were withdrawn from the study as compared to the control group. This result primarily was because of development of systemic disease, which was not attributed to the diet. The study design sought to maximize power by including an ECL and a number of secondary outcome measures. In multicenter trials involving human subjects, an echocardiographic core minimizes variability and increases study power and precision.^15^ Our statistical results do not suggest inadequate power. The *P* values associated with the outcome measures were relatively high, including the *P* value (*P* = .79) for the primary outcome measure. Moreover, results of our secondary analyses were uniformly consistent with the primary finding. Discrepant results between small effectiveness trials and larger confirmatory trials occur across a variety of settings and conditions in human medicine.^16^ The small size of most veterinary trials relative to trials in humans further complicates study design and interpretation.

Our study had some limitations. Design of our study predated quantitative criteria distinguishing dogs with subclinical DMVD. By coincidence, our study and subsequent consensus guidelines^1^ chose to use previously reported LA and LV dimensions^9^ as the basis for study inclusion and definition of DMVD Stage B1, respectively. In light of the guidelines^1^ current at the time of writing, our study population involved dogs with Stage B1 DMVD. The study diets were manufactured at 3 separate plants with 1 plant supplying each of the 3 specific geographic regions. Variations in formula or content of the diets between regions was possible, but our analyses indicate that both the primary and secondary outcomes were consistent across all 3 regions, suggesting the absence of an important effect of manufacturing variations. The composite echocardiographic outcome in our study is a surrogate marker for more clinically relevant events such as onset of CHF or mortality. Heart size is a useful surrogate marker of DMVD progression based on its strong association with risk of future clinical events.^17^ In many instances, drugs or interventions for DMVD have mechanisms of action closely linked to eccentric hypertrophy and cardiac dimensions.^9^, ^18^, ^19^, ^20^ For example, diuretics, positive inotropes, and neurohormonal blocking agents directly affect volume status and contractility and, by extension, heart size. In our study, which involved using diet to change patterns of myocardial substrate usage for energy production, outcome measures involving heart size, although convenient to obtain, might be relatively insensitive. Previous dietary studies in human patients utilized relatively complicated procedures such as serum metabolic profiles and myocardial biopsies.^21^, ^22^, ^23^ These types of data are difficult to obtain in veterinary practice but potentially important in aiding assessment of diet and other interventions targeting the cardiac metabolome.

In conclusion, a specially formulated diet was not associated with significant changes in LAD and LVIDd when fed to dogs with mild subclinical DMVD for 1 year as compared to control diet. Additional studies are needed to better understand the effect of diet and the metabolome in dogs with DMVD.

## CONFLICT OF INTEREST DECLARATION

Drs. Oyama, Scansen, Boswood, and Goldfeder have received honoraria, consulting fees, reimbursement for travel, and grants from Nestle Purina. Qinghong Li is a senior research scientist at Nestle Purina. No other authors declare a conflict of interest.

## OFF‐LABEL ANTIMICROBIAL DECLARATION

Authors declare no off‐label use of antimicrobials.

## INSTITUTIONAL ANIMAL CARE AND USE COMMITTEE (IACUC) OR OTHER APPROVAL DECLARATION

Approved by University of Pennsylvania IACUC, #806367.

## HUMAN ETHICS APPROVAL DECLARATION

Authors declare human ethics approval was not needed for this study.

## Supporting information


**Data S1.** Supporting Information.
