# Supplementary material for: Effect of a specially formulated diet on progression of heart enlargement in dogs with subclinical degenerative mitral valve disease
Source: J Vet Intern Med. 2023 Jul 1;37(4):1323–30. doi: 10.1111/jvim.16796 (PMC10365052; doi:10.1111/jvim.16796)
Supplement: Supplementary file 1 — Data S1. Supporting Information. [file JVIM-37-1323-s001.pdf]

Supplemental Information

Supplemental Table 1. Restricted and allowable concomitant medications

*From EPIC Study (Boswood et al, 2017)

RESTRICTED

**ACE-Inhibitors**	enalapril, benazepril, captopril, fosinopril, imidapril, lisinopril, ramipril
**Angiotensin II receptor blockers**	candesartan, telmisartan
**Antiarrhythmics**	lidocaine, bretylium, flecainide, mexilitine, procainamide, phenytoin, propafenone, quinidine, tocainide, beta-blockers [atenolol, bisoprolol, carvedilol, esmolol, metoprolol, nadolol, propranolol], amiodarone, sotalol, Ca-channel blockers [diltiazem, verapamil], digoxin, digitoxin
**Anticholinergics**	atropine, glycopyrrolate, propantheline
**Beta-blockers**	atenolol, bisoprolol, carvedilol, esmolol, metoprolol, nadolol, propranolol
**Diuretics**	furosemide, hydrochlorothiazide, thiazides, chlorothiazide, torasemide, spironolactone, eplerenone
**Inodilators**	pimobendan, levosimendan, milrinone
**Phosphodiesterase V inhibitors**	sildenafil, tadalafil
**Positive inotropes**	pimobendan, levosimendan, milrinone, isoproterenol, dobutamine, dopamine, digoxin, digitoxin
**Pressor agents**	epinephrine, norepinephrine, phenlyephrine
**Vasodilators**<br><br>**(including nitric oxide donors)**	amlodipine, hydralazine, prazosin, nitroglycerine (even topical), isorbide di-/mononitrate, other nitrates, nitric oxide, sodium nitroprusside, L-arginine
**Dietary supplements**	Fish oil, amino acids, co-enzyme Q10, vitamin and mineral supplements, hawthorn berry
**Other**	iloprost, epoprostenol, bosentan, known cardio toxins e.g. adriamycin

ALLOWABLE

**Bronchodilators and Cough suppressants**	Aminophylline, theophylline, terbutaline, Hydrocodone, butorphanol

Table S1. Baseline biochemical values of the per protocol population.

	Control	Test	P
N	55	46	
ALT	42 (30-72)	39 (27-59)	.21
Hematocrit (%)	47 (5)	49 (5)	.13
Total protein (g/dL)	6.7 (.6)	6.8 (.6)	.35
Creatinine (mg/dL)	0.9 (.7-1.0) (n=54)	0.9 (.8-1.0)	.88
Potassium (mmol/L)	4.5 (4.3-4.7) (n=54)	4.6 (4.3-4.7)	.89
Sodium (mmol/L)	147 (146-148) (n=54)	147 (145-149)	.83

ALT, alanine transaminase

Table S2. Change in biochemistry and body weight from baseline to day 365 for the per protocol population.

	Control	Test	P
N	55	46	
ALT	-9 (-21 to 4) (n=54)	5 (-3 to 15) (n=45)	<.001
Hematocrit (%)	1 (-2 to 2) (n=43)	-1 (-4 to -.5) (n=40)	.002
Total protein (g/dL)	-0.4 (.6)	0.1 (.6)	.37
Creatinine (mg/dL)	0 (-.1 to .1) (n=53)	-0.1 (-.1 to .1)	.049
Potassium (mmol/L)	0.1 (-.1 to .4) (n=53)	-0.1 (-.3 to .2)	.036
Sodium (mmol/L)	-1 (-2 to 1) (n=53)	-1 (-3 to 0)	.072
Body weight (kg)	.1 (.6) (n=54)	0 (.7)	.74

ALT, alanine transaminase

Supplemental Information:
Diet tolerability scale for use during run-in period

Owner scoring system for palatability:

[1] My dog eats the study diet with a similar degree of enthusiasm, completeness, and overall acceptance as its regular diet

[2] My dog eats the study diet with a noticeably lower degree of enthusiasm, completeness, and overall acceptance as its regular diet

[3] My dog will not eat the study diet or only eats it with an extremely low degree of enthusiasm, completeness, and overall acceptance as its regular diet

Owner scoring system for tolerance:

[1] My dog appears unchanged with respect to gastrointestinal (i.e., vomiting, diarrhea) allergic (i.e., itching, skin issues), or other problems as compared to its normal diet

[2] My dog appears mildly to moderately worse with respect to gastrointestinal (i.e., vomiting, diarrhea) allergic (i.e., itching, skin issues), or other problems as compared to its normal diet

[3] My dog appears extremely worse with respect to gastrointestinal (i.e., vomiting, diarrhea) allergic (i.e., itching, skin issues), or other problems as compared to its normal diet

Supplemental Information:
CARMINE Echo Guidelines
Echocardiographic core laboratory
BA Scansen, 15 September 2017

1. **Echocardiographic Images (6+ cardiac cycles of each):**
   - 1.1 Right parasternal short axis (RPSAX) M-mode of the left ventricle (LV) at the level of the tips of the papillary muscles for LV chamber dimensions and %FS
     - 1.1.1. Sweep speed = 100 mm/sec
   - 1.2 RPSAX 2D cine loop of the left atrium and aorta for LA:Ao by Swedish method
   - 1.3 Right parasternal long axis RPLAX 2D cine loop of the 4-chamber view with color Doppler over the mitral valve
   - 1.4 RPLAX 2D cine loop of the 4-chamber view with attention to capturing the left atrium maximal diameter
   - 1.5 RPLAX 2D cine loop of left ventricle, outflow tract and aorta with attention to capturing sagittal plane of aortic valve annulus
   - 1.6 Left apical 4-chamber view with color Doppler over the mitral valve
   - 1.7 Left apical 4-chamber view with spectral Doppler images of diastolic mitral inflow
     - 1.7.1 Sample volume = 2 to 3 mm; sweep speed = 100 mm/sec
   - 1.8 Image format is DICOM

2. Image Specific Guidelines

2.1 RPSAX M-mode of LV

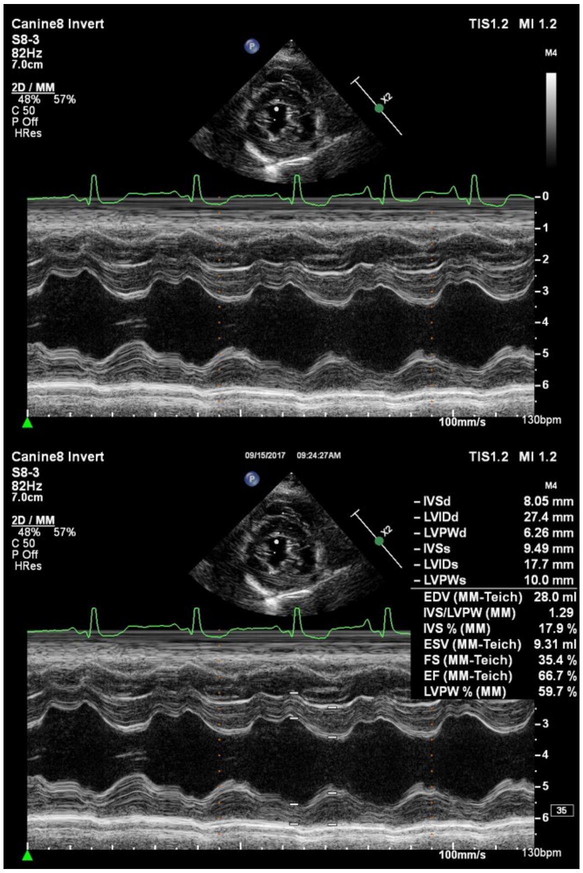

2.1.1 Aim for upright LV with cursor bisecting PPMs
2.1.2 Position probe sufficiently dorsal on thorax to include RV lumen
2.1.3 At level of valve chordae, just below visualization of MV leaflets
2.1.4 Sweep speed set at 100 mm/sec

2.2 RPSAX LA:Ao

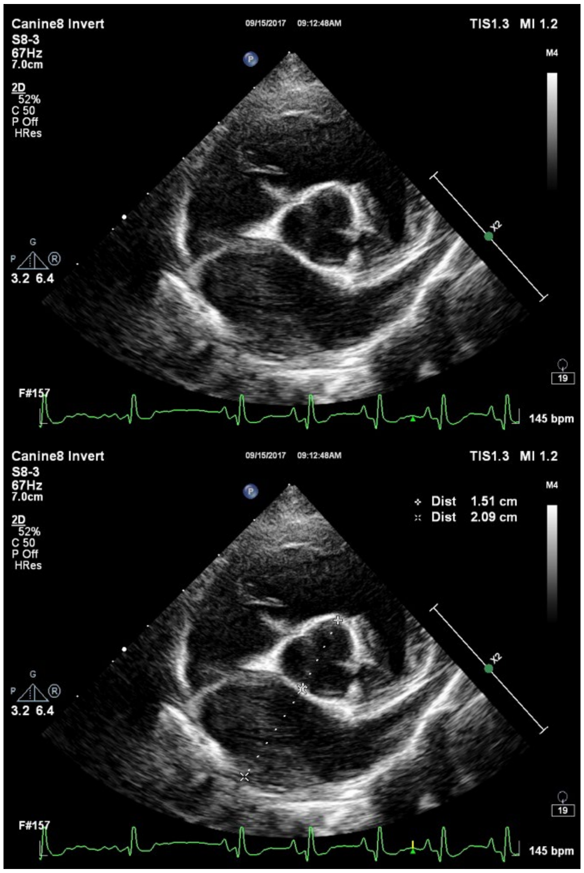

2.2.1 Optimize body of LA with left auricle partially/fully visible
2.2.2 Avoid PA branches overlying LA
2.2.3 Ao sinuses should be symmetrical
2.2.4 Optimize imaging at onset of diastole; measurement is made at first frame after aortic valve closure.
2.2.5 Measure Ao from inner edge (at blood-tissue interface) of mid-point at curvature of the right adjacent sinus. Extend line along commissure of left and non-adjacent sinuses to the site of merger between aortic wall, left adjacent sinus and non-adjacent sinus.
2.2.6 Measure LA beginning at same point that Ao line ended, parallel to Ao measurement, to the inner edge (blood-tissue interface) of the left atrial wall. Extrapolate inner edge of wall if pulmonary venous ostium enters at desired site of measurement.

2.3 RPLAX 4-chamber LA

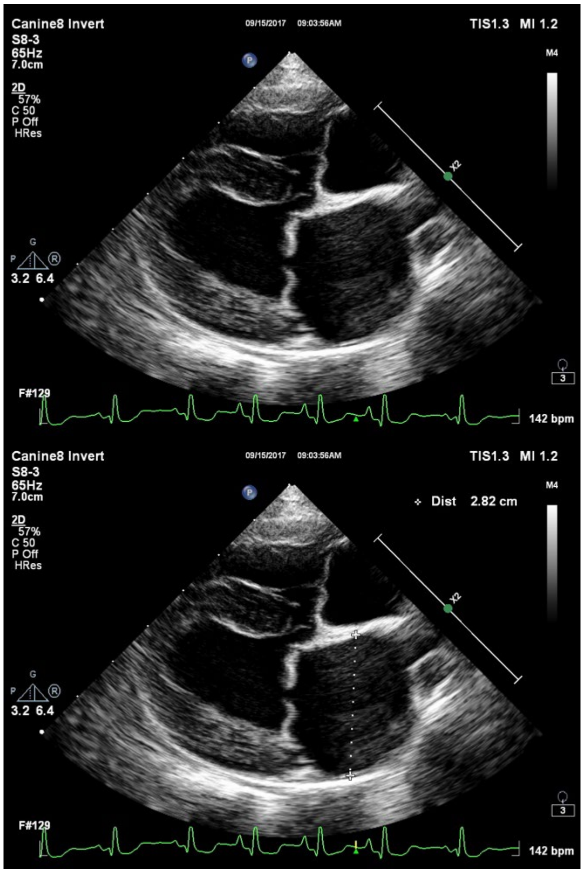

2.3.1 4-chamber view horizontal across screen
2.3.2 Optimize so interatrial septum is clearly delineated and right caudal pulmonary vein can be seen entering LA along IAS
2.3.3 Avoid having left auricle in the far field
2.3.4 Timing of measurement is one frame prior to mitral valve opening (end ventricular systole).
2.3.5 Measurement is made from blood-tissue interface of interatrial septum, bisecting body of LA, to blood-tissue interface of posterior LA wall. Line of measurement is parallel to mitral valve annulus.

2.4 RPLAX 4-chamber AoV

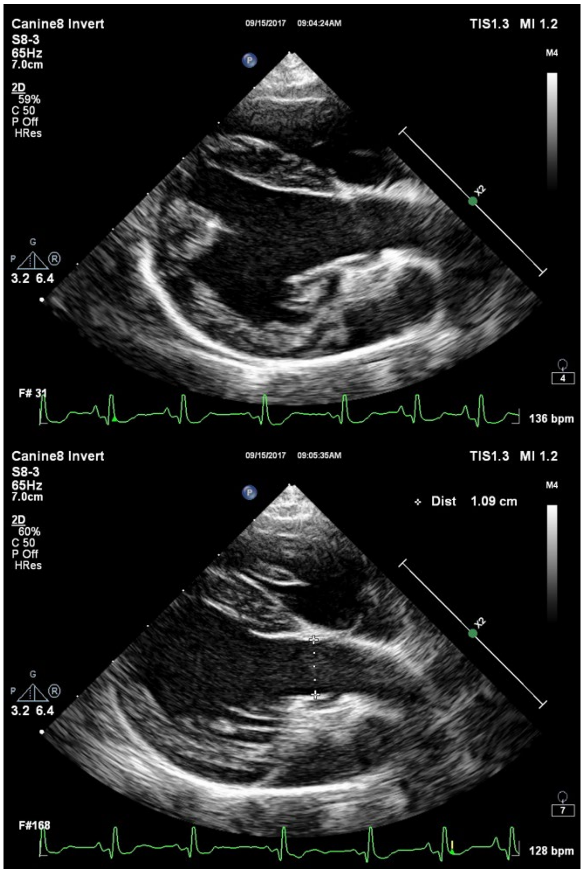

2.4.1 5-chamber view of LVOT horizontal across screen
2.4.2 Optimize to mid-line (sagittal) of aortic root
2.4.3 Desire to see opening and closing of near-field and far-field Ao valve leaflets
2.4.4 Optimal image includes parallel walls of ascending aorta at maximal diameter
2.4.5 Timing of measurement is 2-3 frames after onset of systole with aortic valve leaflets parallel to one another
2.4.6 Measurement is made at mid-point of near-field leaflet at blood-tissue interface perpendicular to aortic root to the mid-point of far-field leaflet at blood-tissue interface.

2.5 Left Apical 4-chamber

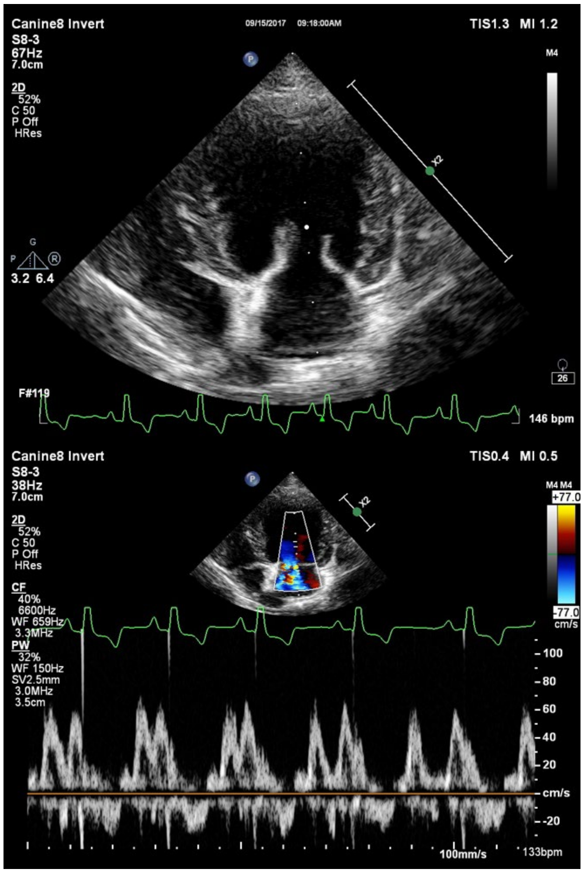

2.5.1 Upright LV parallel to cursor
2.5.2 Cursor positioned at open tips of mitral valve with 2 to 3 mm sample volume
2.5.3 2-D guiding image is frozen during spectral acquisition
2.5.4 Sweep speed is set at 100 mm/sec

3. File Structure

3.1 Within each site folder, create folders for each patient enrolled. These patient folders will include all data saved for this patient and are labeled as: Site_Patient#_Name

3.2 Within each patient folder, created folders for each echo study that is uploaded. Folders for echo studies should be labeled as Site_Patient#_Name_EchoDay.

3.3 As examples…

3.3.1 The echo images for the 180 day study from patient enrolled at Penn that was assigned study number P-3 from the site's randomization table would be found in the folder: Penn\Penn_P3_FluffySmith\Penn_P3_FluffySmith_Echo180d

3.3.2 The baseline echo images for the patient enrolled at RVC that was assigned study number R-15 from the site's randomization table would be found in the folder: RVC\RVC_R15_JaneDoe\RVC_R15_JaneDoe_Echo0d

3.3.3 The echo images for the 365 day study from the patient enrolled at USP that was assigned study number B-8 from the site's randomization table would be found in the folder: USP\USP_B8_BrunoOrtiz\USP_B8_BrunoOrtiz_Echo365d
